# Detection of Aminoglycoside Resistant Bacteria in Sludge Samples From Norwegian Drinking Water Treatment Plants

**DOI:** 10.3389/fmicb.2019.00487

**Published:** 2019-03-13

**Authors:** Ingvild F. Ullmann, Hege S. Tunsjø, Monica Andreassen, Kaare Magne Nielsen, Vidar Lund, Colin Charnock

**Affiliations:** ^1^Department of Life Sciences and Health, OsloMet – Oslo Metropolitan University, Oslo, Norway; ^2^Department of Zoonotic, Food- and Waterborne Infections, Norwegian Institute of Public Health, Oslo, Norway

**Keywords:** antimicrobial resistance (AMR), water treatment, sludge, agriculture, drinking water, environmental bacteria

## Abstract

Through a culture-based approach using sludge from drinking water treatment plants, this study reports on the presence of aminoglycoside resistant bacteria at 23 different geographical locations in Norway. Sludge samples are derived from a large environmental area including drinking water sources and their surrounding catchment areas. Aminoglycoside resistant bacteria were detected at 18 of the sample sites. Only five samples did not show any growth of isolates resistant to the selected aminoglycosides, kanamycin and gentamycin. There was a statistically significant correlation between the numbers of kanamycin and gentamycin resistant bacteria isolated from the 23 samples, perhaps suggesting common determinants of resistance. Based on 16S rRNA sequencing of 223 aminoglycoside resistant isolates, three different genera of *Bacteroidetes* were found to dominate across samples. These were *Flavobacterium, Mucilaginibacter* and *Pedobacter.* Further phenotypic and genotypic analyses showed that efflux pumps, reduced membrane permeability and four assayed genes coding for aminoglycoside modifying enzymes AAC(6′)-Ib, AAC(3′)-II, APH(3′)-II, APH(3′)-III, could only explain the resistance of a few of the isolates selected for testing. *aph(3*′*)-II* was detected in 1.6% of total isolates, *aac(6*′*)-Ib* and *aph(3*′*)-III* in 0.8%, while *aac(3*′*)-II* was not detected in any of the isolates. The isolates, for which potential resistance mechanisms were found, represented 13 different genera suggesting that aminoglycoside resistance is widespread in bacterial genera indigenous to sludge. The present study suggests that aminoglycoside resistant bacteria are present in Norwegian environments with limited anthropogenic exposures. However, the resistance mechanisms remain largely unknown, and further analyses, including culture-independent methods, could be performed to investigate other potential resistance mechanisms. This is, to our knowledge, the first large scale nationwide investigation of aminoglycoside resistance in the Norwegian environment.

## Introduction

The Global Action Plan on Antimicrobial Resistance presented by the WHO in 2015, states that the “one health” perspective is an important approach in tackling the worldwide challenge represented by antimicrobial resistance (AR) (WHO, 2015). The “one health” concept stresses not only the importance of human and veterinary medicine, but also agriculture and environmental aspects of AR. In regards to the environmental dimensions of AR, there is a need for a more detailed understanding of the prevalence of resistant bacteria in natural environments, the resistance mechanisms they use and the phenotypic variability they represent (Larsson et al., 2018). The purpose of this study was to investigate the occurrence of aminoglycoside resistance in sludge from drinking water treatment plants (DWTPs) in Norway. This material is important as it derives from large and in many cases relatively pristine environments (lakes and catchment areas) and because sludge often enters the municipal waste disposal system from where it can be spread to other areas. Increasingly in Norway, sludge based products are used as organic fertilizers.

According to the Norwegian Surveillance Program for antimicrobial resistance (NORM-VET), the use of aminoglycosides (AG) in veterinary and clinical use has been below 10% of the total sales (5,450 kg) of antibiotics since 2016 (NORM/NORM-VET, 2016). Thus, the expected levels of pollution of AG from agriculture and clinical use in Norwegian environments should be low. AG are potent, broad-spectrum, bactericidal antibiotics that act primarily by binding to the A-site of 16S rRNA and thereby disrupting protein synthesis (Krause et al., 2016). AG are particularly effective against infections caused by aerobic, Gram-negative bacteria (Hermann, 2007). Mechanisms of resistance to AG are diverse, however, the most widespread strategy used by clinical isolates is the inactivation of these antibiotics by AG-modifying enzymes, AME (Garneau-Tsodikova and Labby, 2016). Additional mechanisms providing resistance include efflux pumps (Fernandez and Hancock, 2012) such as MexXY in *Pseudomonas aeruginosa* (Morita et al., 2012), and methyltransferases, which modify ribosomes (Doi et al., 2016). Furthermore, mutations in the ribosomal target of AG (Ying et al., 2018) as well as the natural barrier represented by the Gram-negative outer membrane (OM) in synergy with efflux pumps (Krishnamoorthy et al., 2017), can also contribute to resistance. With regards to the latter, intracellular accumulation of antibiotics can be further decreased by mutations altering OM permeability to AG (see Garneau-Tsodikova and Labby, 2016. for a comprehensive review of AG resistance mechanisms).

AG resistance genes are often associated with mobile genetic elements (Galimand et al., 2003; Yokoyama et al., 2003; Golebiewski et al., 2007). AAC(6’) enzymes, by far the most common AME, are present in Gram-negative as well as Gram-positive bacteria. The genes have been found on plasmids as well as on chromosomes, and are often part of mobile genetic elements (Tolmasky, 2000; Centron and Roy, 2002; Soler Bistue et al., 2008). AAC(6′)-Ib is the most prevalent and clinically relevant AME and a large number of variants exist. The enzyme is present in over 70% of AAC(6′)-producing Gram-negative isolates (Vakulenko and Mobashery, 2003). AAC(3′)-II is also widely distributed among Gram-negative bacteria where it is typically associated with resistance to tobramycin and gentamycin. Genetic determinants of this enzyme class have been found on transposons, plasmids and as chromosomal genes (Vakulenko and Mobashery, 2003; Ramirez and Tolmasky, 2010). The AG resistance genes *aph(3*′*)-II* (*nptII*) and *aph(3*′*)-III* (*nptIII*) encode AG phosphotransferases (APH). While NPTII mainly inactivates kanamycin and neomycin, NPTIII additionally phosphorylates amikacin. NPTIII appears to be the most prevalent APH in Gram-positive bacteria and shows the broadest spectrum of activity of the family (Fong and Berghuis, 2002; Becker and Cooper, 2013). Both APH genes are associated with mobile genetic elements (Beck et al., 1982). *npt-*genes have also received much attention as they are the most used antibiotic resistance markers (ARM) in plant gene technology (Miki and McHugh, 2004; Rosellini, 2012). There is a natural concern that genetically modified (GM) plants might shed ARM to the soil, ultimately leading to a rise in resistant bacterial phenotypes. At the time of writing Norway is a GMO-free region.

Given their importance in human and veterinary medicine and their presence in GM crops, surprisingly few studies exist documenting the presence, spread and concentrations of AG and genetic determinants of AG-resistance in the environment. Woegerbauer et al. (2015) investigated the baseline values of *aph(3*′*)-IIa* and *aph(3)-IIIa* in GMO-pristine agricultural soils in Austria, finding 6 and 85% of fields to be positive for the presence of these genes, respectively. However, the genes were present in only 1.8% (*aph(3*′*)-IIIa*) and essentially zero% (*aph(3*′*)-IIa*) of kanamycin resistant strains (Woegerbauer et al., 2015). Given the lack of other studies for comparison, the generality of these results remains uncertain. However, the Austrian authors suggested that the load of *aph(3*′*)-IIa* was so low that it fulfills all requirements to be considered as an environmental pollutant if released through anthropogenic activities into the tested ecosystems. Given the low baseline values, release of these ARM genes into pristine environments would cause a significant increase above the natural baseline levels, warranting their status as pollutants (Martinez, 2009). More importantly, resistance genes may spread and be preserved in the environment, in contrast to antibiotics which degrade over time (Martinez, 2009).

The present study determines the presence of AG-resistant bacteria and resistance genes in sludge samples produced at Norwegian drinking water treatment plants (DWTPs). The choice of AG resistance genes assayed for was based on their clinical significance *(aac(6*′*)-Ib, aac(3*′*)-II)* and on the availability of data from similar studies for purposes of comparison (*aph(3*′*)-II; aph(3*′*)-III*). Gentamycin resistance was chosen for investigation, as this AG remains an important treatment option for sepsis in Norway. Furthermore, gentamycin is the most widely used aminoglycoside in the country (NORM/NORM-VET, 2016). Kanamycin was included specifically because of the connection between the *nptII* gene used in GMO and kanamycin resistance. Furthermore, other studies on kanamycin resistance in environmental isolates are available for comparison (Woegerbauer et al., 2015).

## Materials and Methods

### Sample Collection and Sample Sites

**FIGURE 1:**
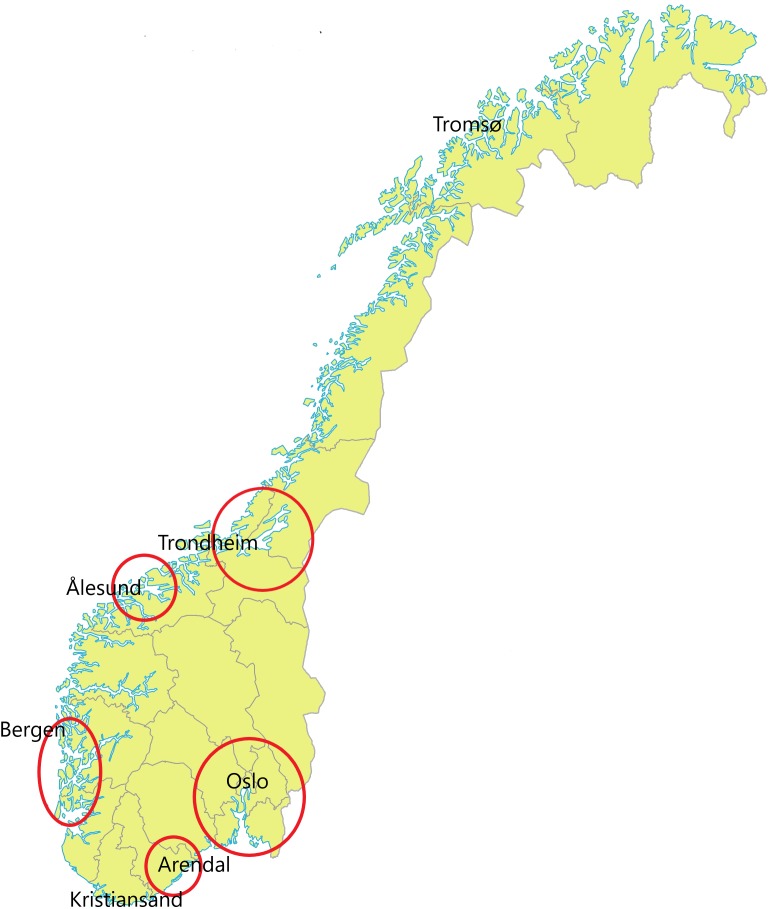
Map of Norway (Statens Kartverk, 2016) with red marks showing the geographical locations of the water treatment plants where samples were collected.

Twenty-three DWTPs employing conventional water treatment (coagulation-filtration) from different geographical locations in Norway (Figure 1) that utilize surface water, contributed sludge samples to the project. All samples were collected in the period June 2016 to November 2017. DWTPs were provided with sampling equipment, and the sludge samples were collected by employees at each DWTP according to a protocol developed by the Norwegian Institute of Public Health. In brief, two 50 ml screw-cork tubes were filled with sludge material prior to dewatering of the sludge. Plastic gloves were used during the sampling procedure to minimize contamination. Samples were shipped with cooling elements to the laboratory overnight. The procedure ensured DNA extraction and cultivation within 24 h of sample collection.

### Cultivation of Bacteria

Approximately five grams of wet weight sludge, taken directly from sample tubes was added to 45 ml of 10% tryptic soy broth (TSB) (Sigma–Aldrich: Merck, Darmstadt, Germany) in 50 ml tubes and set for shaking at 250 rpm for 30 min to release bacteria from organic material. Thereafter, samples were allowed to stand for 10 min to allow large particles to settle. A sample was withdrawn from the upper aqueous phase from each tube, and a dilution series of 10^-1^ to 10^-7^ in TSB was set up in two parallels. Then, 100 μl of each dilution was plated onto: R2A media (Oxoid, Basingstoke, United Kingdom) without antibiotics, R2A with 32 μg/ml of Kanamycin (Sigma–Aldrich) and R2A with 8 μg/ml Gentamycin (Sigma–Aldrich). The antibiotic concentrations were chosen to correspond to break point values for intermediate resistance for *Enterobacteriaceae* presented in the CLSI (Wayne, PA, United States) standard M100 (CLSI, 2017). Cycloheximide (Sigma–Aldrich) 50 μg/ml and nystatin (Sigma–Aldrich) 10 μg/ml were added to plates to inhibit the growth of fungi. Plates were incubated at 22°C ± 2°C for 14 days to allow for the growth of slow-growing bacteria. Colonies growing on plates containing antibiotic were picked and stored at -80°C for further characterization and resistance determination.

### DNA Extraction From Antibiotic Resistant Bacteria

DNA was extracted from 245 antibiotic resistant bacterial isolates by boiling for 10 min followed by centrifugation to remove cell debris. The supernatant, containing bacterial DNA in nuclease free water, was transferred to a new 1.5 ml microcentrifuge tube and stored at -20°C for later use as template in PCR reactions. This method of DNA extraction was chosen in order to harvest both plasmid and chromosomal DNA.

### Sanger Sequencing of the 16S rRNA Gene

The 16S rRNA gene from 245 isolates that showed resistance to either kanamycin or gentamycin was amplified using LongAmp^®^ *Taq* DNA Polymerase (NEW ENGLAND BioLabs^®^Inc., Ipswich, MA, United States). Primers used were 27F (Lane, 1991) (5′ TGAAGAGTTTGATCATGGCTCAG 3′) and modified 805R (Tanner et al., 1998) (5′ GGCGTGGACTACCAGGG 3′) amplifying the V3 and V4 regions of the 16S rRNA gene. The PCR cycling conditions were: hot start 95°C 60 s, 30 cycles of denaturation 95°C 20 s, annealing 57°C 30 s, elongation 65°C 2 min, followed by a 5 min final elongation step at 65°C. To every reaction, 2 μL template was added. Amplicon size was determined using QIAxcel Capillary electrophoresis (QIAGEN, Hilden, Germany), while amplicon concentrations were measured using Qubit^TM^ dsDNA BR Assay Kit (ThermoFisher Scientific, Waltham, MA, United States). The 16S rRNA amplicons of 245 colonies were submitted to GATC-Biotech (Eurofins Genomics) for PCR purification and single-read SUPREMERUN 96 barcode Sanger sequencing (ABI 3730xl DNA analysis systems). Returned ABI files were quality controlled, trimmed and FASTA formatted using Sequencher (Gene Code Cooperation) before taxonomy classification was performed in SILVA (Pruesse et al., 2012). Classified sequences were annotated to the appropriate sample site, and the open source software R, (R Core Team, 2017) was used to create visual representations of these findings primarily using functions in the R package ggplot2. A phylogenetic tree based on 16S rRNA amplicon sequencing was produced with RAxML 8.2.9 (Stamatakis, 2014) in SILVA, and edited in the online tool Interactive tree of life (iTOL) version 4.3 by European Molecular Biology Laboratory (EMBL) (Ciccarelli et al., 2006). All sequencing data can be found publicly available at https://doi.org/10.6084/m9.figshare.7053458.v1.

### Genotypic Analysis of Aminoglycoside Resistance Determinants

The 245 kanamycin and gentamycin resistant isolates were subjected to a multiplex PCR assay to screen for four aminoglycoside resistance genes (Table 1). Primers (Table 1) used in this assay were based on Hu et al. (2013) and Woegerbauer et al. (2014). To validate primer performance and specificity, positive and negative controls for each target gene were analyzed in separate assays and in a multiplex assay. Control strains were: *aac(3)-II: E. coli* K53-1, *aac(6*′*)-Ib: E. coli* A3-21 (Haldorsen et al., 2014), a*ph(3*′*)-II nptII: Acinetobacter baylyi* JV31, *aph(3*′*)-III nptIII* (de Vries et al., 2003). QIAGEN^®^ Multiplex PCR Kit (QIAGEN) was used according to the manufacturer’s protocol with a reaction volume of 25 μl including 2 μl of template DNA. PCR was performed as follows: hot start 95°C 15 min, 30 cycles of denaturation 94°C 30 sec, annealing 55°C 90 s, elongation 72°C 60 s. A final elongation step was performed at 72°C for 10 min. Results were analyzed and correct amplicon sizes were confirmed using QIAxcel Capillary electrophoresis (QIAGEN) according to manufacturer’s default settings. All PCR reactions were performed in triplicate.

**Table 1:** Primer information for multiplex PCR assay.

Target gene	Gene size (bp)	Primer	Amplicon size	Sequence (5′– > 3′)
*aph(3*′*)-III nptIII*	795	npt3_F	82	ACATATCGGATTGTCCCTATACGAA
		npt3_R		TCGGCCAGATCGTTATTCAGTA
*aph(3*′*)-II nptII*	795	npt2_F	129	GATCTCCTGTCATCTCACCTTGCT
		npt2_R		TCGCTCGATGCGATGTTTC
*aac(3*′*)-II*	877	aac(3′)-II_F	274	ACTGTGATGGGATACGCGTC
		aac(3′)-II_R		CTCCGTCAGCGTTTCAGCYA
*aac(6*′*)-Ib*	472	aac(6′)-Ib_F	188	CTGTTCAATGATCCCGAGGT
		aac(6′)-Ib_R		TGGCGTGTTTGAACCATGTA


*Primers were based on (Hu et al., 2013) and (Woegerbauer et al., 2014)*.

### Phenotypic Analysis of Aminoglycoside Resistance

#### Minimum Inhibitory Concentration (MIC) Assay

The MIC of gentamycin and kanamycin against bacterial isolates representing every taxa identified by Sanger sequencing were measured based on a modification of the standard procedures given in CLSI (M100 2014). Five to ten different isolates from the three most abundant bacterial genera were analyzed. Twofold dilution series of gentamycin and kanamycin in cation-adjusted Mueller Hinton Broth (MH) (Oxoid) were set up in microtiter plates with final concentrations ranging from 256 to 4 mg/L. Four to ten colonies from each isolate were homogenized in 0.9% NaCl to achieve a final concentration equivalent to 0.5 McFarland. The bacterial suspensions were further diluted 1:150 in MH before 50 μl was transferred to the microtiter wells containing 50 μl MH with gentamycin or kanamycin. The assay was further modified to suit slow growing environmental bacteria. The temperature was reduced from 35°C to 28°C, and the incubation time was increased from 16–20 h to 96 h. Growth was registered visually every day for 4 days. *Escherichia coli* strain ATCC 25922 was used as a quality control reference for determination of QC breakpoints as recommended by CLSI (2017).

### Efflux Pumps and Outer Membrane Resistance Assay

To investigate the possible contribution of efflux pumps and outer membrane permeability on the observed MICs, bacteria were grown in parallel in the same antibiotic dilution series as described above, but in the presence of 50 μM carbonyl cyanide m-chlorophenyl hydrazone (CCCP) (Sigma–Aldrich), (Gad et al., 2011) or 1 mM EDTA. CCCP is a H^+^ conductor that inhibits various transporters driven directly or indirectly by an electrochemical potential of H^+^ (Morita et al., 1998). EDTA is capable of chelating a variety of divalent cations (e.g., Mg^2+^, Ca^2+^) and permeabilizes an outer membrane of Gram-negative bacteria (Alakomi et al., 2006). MIC values were subsequently recorded and compared with the values in the absence of these agents.

### Statistical Analysis

The range, mean and standard deviation of CFU/mg were calculated for kanamycin- and gentamycin-resistant isolates. Data sets of log-transformed CFU/mg were compared using the paired Students *t*-test.

## Results

### Aminoglycoside Resistant Bacterial Isolates

Samples from 18 of the in total 23 DWTPs investigated, gave visible colonies on gentamycin and/or kanamycin containing agar, indicating the presence of resistant bacteria (Table 2). Differences of more than an order of magnitude in the CFU/mg of resistant bacteria in sludge samples from different DWTs were seen. For example, the concentration of Km-resistant bacteria at DWTP_6 was almost 50 × that of DWTP_2. Sludge samples from eight DWTPs gave no growth of kanamycin resistant bacteria. The corresponding number for gentamycin was six DWTPs. Four samples produced growth on plates with only one or the other antibiotic (Table 2). Only one of the collected samples failed to produce colonies on any of the R2A media, with or without antibiotics. Comparison of the data sets using the paired *t*-test (Table 3) showed that the difference between CFU/mg on kanamycin- and gentamycin- containing agars was not statistically significant.

**Table 2:** Norwegian water treatment plants investigated in this study.

Plant	County	Water source	% farming in catchment area	R2A CFU/mg	Km CFU/mg	Gm CFU/mg	%Km resistant bacteria	%Gm resistant bacteria
DWTP_1	Oslo	Lake	11	1.15E+06	7.36E+04	N/A	6	N/A
DWTP_2	Oslo	Lake	0	2.85E+05	6.20E+03	N/A	2	N/A
DWTP_3	Østfold	Lake	22	2.08E+06	1.44E+05	N/A	7	N/A
DWTP_4	Akershus	Lake	0	3.08E+05	1.14E+04	N/A	4	N/A
DWTP_5	Hordaland	Lake	0	2.64E+05	1.10E+04	<5,10E+03	4	–
DWTP_6	Hordaland	Lake	0	1.67E+06	2.93E+05	4.27E+04	18	3
DWTP_7	Hordaland	Lake	0	3.93E+05	4.59E+04	5.10E+04	12	13
DWTP_8	Østfold	River	50	7.21E+05	N/A	3.66E+05	N/A	51
DWTP_9	Hordaland	Lake	0	8.25E+04	<5,50E+03	1.10E+04	–	13
DWTP_10	Trøndelag	Lake	10	1.28E+05	<5,10E+03	<5,10E+03	–	–
DWTP_11	Akershus	Lake	15	6.26E+05	<5,40E+03	5.40E+04	–	9
DWTP_12	Møre og Romsdal	Lake	0	6.48E+04	<5,40E+03	<5,40E+03	–	–
DWTP_13	Aust-Agder	Lake	0	1.17E+05	<5,10E+03	<5,10E+03	–	–
DWTP_14	Akershus	Lake	0	3.39E+05	<5,30E+03	1.06E+04	–	3
DWTP_15	Telemark	Lake	0	1.06E+06	1.80E+05	3.92E+05	17	37
DWTP_16	Østfold	River	0	7.95E+05	2.54E+05	3.02E+05	32	38
DWTP_17	Rogaland	Lake	0	2.32E+05	5.94E+04	4.86E+04	26	21
DWTP_18	Akershus	River	5	1.07E+06	3.31E+05	3.36E+05	31	31
DWTP_19	Akershus	Lake	0	4.43E+05	3.78E+04	2.70E+04	8	6
DWTP_20	Aust-Agder	River	8	2.44E+05	3.12E+04	4.68E+04	13	19
DWTP_21	Akershus	Lake	0	5.29E+05	2.70E+04	2.16E+04	5	4
DWTP_22	Vestfold	Lake	2	3.89E+05	<5,80E+03	<5,80E+03	–	–
DWTP_23	Trøndelag	Lake	10	<5,70E+03	<5,70E+03	<5,70E+03	–	–


*Km, kanamycin; Gm, gentamycin*.

**Table 3:** *Pairwise comparisons of CFU/mg obtained with and without kanamycin (Km) and gentamycin (Gm)*.

	Km CFU/mg	Gm CFU/mg
Mean	7,04E+04	9,22E+04
Standard deviation	9,97E+04	1,35E+05
Sample size	22	19
Range	3,26E+05	3,87E+05
*t*-test two-tailed P value compared to R2A CFU/mg	<0.0001	<0.0001
*t*-test two-tailed P value comparing Km CFU/mg and Gm CFU/mg	0.37	0.37


^∗^*Paired t-test was performed on the log CFU/mg values presented in Table 2*

### Bacterial Identification Based on 16S rRNA Sequencing Data

**FIGURE 2:**
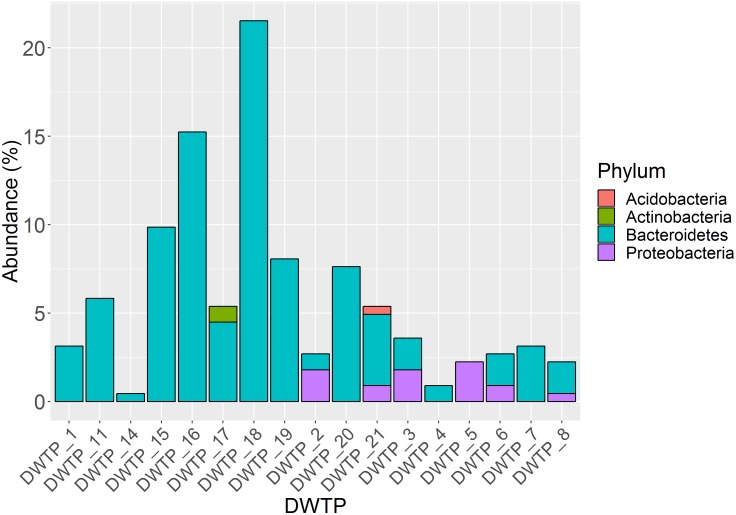
Proportion of four different phyla among 223 identified isolates and their distribution at 17 DWTPs

**FIGURE 3:**
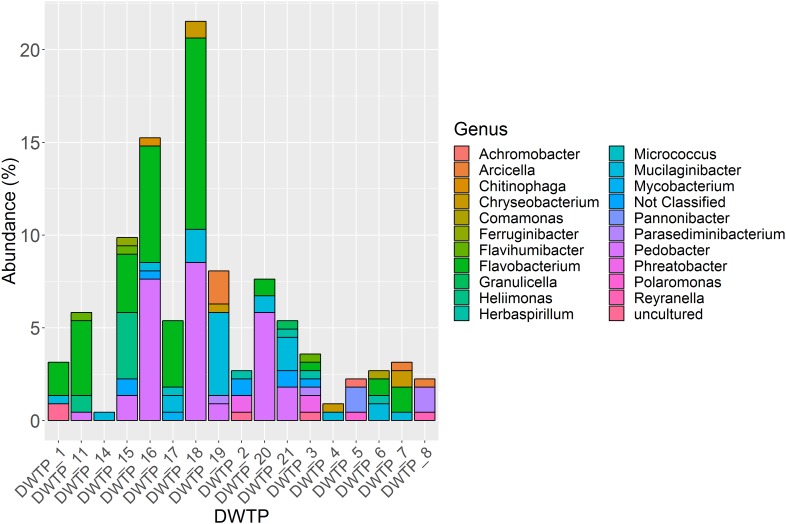
Proportion of 21 different genera among 223 identified isolates and their distribution at 17 DWTPs. Eight isolates were not identified at genus level and are thus noted as not classified. The three main genera represented are *Flavobacterium* (bright green), *Pedobacter* (bright purple) and *Mucilaginibacter* (turquoise).

In total, 245 aminoglycoside resistant isolates from 17 DWTPs were chosen for further analysis based on unique colony morphology. 16S rRNA amplicon sequencing of the 245 antibiotic resistant bacterial isolates identified 223 to the family level, and 215 to genus level. In total, 20 different genera were identified. These were distributed across ten different orders belonging to three phyla of Gramm-negative bacteria and the *Actinobacteria*. Of the total 223 resistant isolates, the phylum *Bacteroidetes* dominated numerically with 202 isolates, accounting for 90% of the identified bacteria. Eighteen isolates (8%) were identified as *Proteobacteria*, while two (0.9%) isolates of the phyla A*ctinobacteria* and one (0.5%) *Acidobacteria* isolate were detected. The relative abundance of these four phyla and their distribution among the different DWTPs is shown in Figure 2. *Bacteroidetes* was detected at all DWTPs except for DWTP_5, where only *Proteobacteria* were found. Furthermore, of the total number of aminoglycoside resistant isolates subjected to 16S rRNA sequencing, 25% of these were *Bacteroidetes* isolated from DWTP_18. The genera *Flavobacterium, Mucilaginibacter* and *Pedobacter*, all of which belong to the *Bacteroidetes* phylum, showed the highest prevalence among the identified isolates. In addition, these three genera were the most widely distributed of all taxa detected as illustrated in Figure 3. *Flavobacterium* made up 30% of the total number of isolates and was detected at ten out of 17 DWTPs, whereas 24% were *Pedobacter* detected at seven different DWTPs. *Mucilaginibacter* was detected at 11 different DWTPs showing the widest distribution of the three genera, but at a lower total proportion (12%) than *Flavobacterium* and *Pedobacter.* There were eight resistant isolates that could not be identified to genus level. These are noted as Not Classified (dark blue) in Figure 3. Two of these isolates belong to the family *Chitinophagaceae*, another two to the *Sphingobacteriaceae* and one isolate to the *Xanthobactereaceae.* The three remaining isolates could only be identified to the order level as *Sphingobacteriales.*

#### Detection of Aminoglycoside Resistance Genes by Multiplex PCR

In total, 245 kanamycin and/or gentamycin resistant isolates were tested using a multiplex PCR assay for the presence of four genes coding for aminoglycoside resistance (see “Materials And Methods” section). Only seven isolates were found to be positive for one or more of the target genes (Table 4). The seven isolates could be mapped to six different genera, and six different DWTPs. Out of the total 245 isolates that were analyzed, *aph(3*′*)-II/nptII* was detected in only 1.6%, whereas 0.8% of the isolates were positive for *aac(6*′*)-Ib* and *aph(3*′*)-III/nptIII* (Table 5).

**Table 4:** Isolates PCR positive for one or more of the target genes.

Isolate	*aac(6*′*)-Ib*	*aac(3*′*)-II*	*aph(3*′*)-II (nptII)*	*aph(3*′*)-III (nptIII)*	Family/Genus	DWTP
B2_33	neg	neg	pos	neg	*Parasediminibacterium*	DWTP_3
B2_35	neg	neg	pos	neg	*Herbaspirillum*	DWTP_3
B5_9	pos	neg	pos	neg	*Heliimonas*	DWTP_11
B6_40	neg	neg	neg	pos	*Pedobacter*	DWTP_16
B6_64	pos	neg	neg	neg	*Mycobacterium*	DWTP_17
B7_61	neg	neg	neg	pos	*Pedobacter*	DWTP_18
B8_56	neg	neg	pos	neg	*Xanthobacteraceae*	DWTP_21


**Table 5:** PCR based detection of genes coding for aminoglycoside modifying enzymes.

Target gene	Number of PCR -positive isolates out of 245 isolates tested	PCR positive isolates of 245 tested isolates, %
*aac(6*′*)-Ib*	2	0.8
*aac(3*′*)-II*	0	0
*aph(3*′*)-III nptIII*	2	0.8
*aph(3*′*)-II nptII*	4	1.6


### Investigation of Efflux Pumps and Membrane Permeability

MIC values were in the range of < 4 mg/L to > 256 mg/L (Table 6). Additional isolates of *Pedobacter* and *Flavobacterium* were included in the assay, as these were the genera detected most frequently in this study. In the presence of CCCP, kanamycin MIC values were reduced for four isolates, while three isolates had a reduction in gentamycin MIC values (Table 6). The four isolates showing a reduction in kanamycin MIC values belong to the genera *Mucilaginibacter, Pannonibacter, Pedobacter* and *Reyranella*. Even though several different isolates from the genus *Pedobacter* were tested in this assay, only one was affected by CCCP. The three isolates in which gentamycin MIC values were reduced in the presence of CCCP, belonged to the genera *Flavihumibacter, Flavobacterium* and *Mycobacterium*. When EDTA was added to the wells, nine isolates showed reduced MIC values for kanamycin, and eight for gentamycin (Table 6). Both kanamycin and gentamycin MIC values remained unchanged for sixteen isolates in the presence of either CCCP or EDTA. In order to assess the stability of the two antibiotics in this set up, the negative control *E. coli* ATCC 25922 was monitored throughout the length of the experiment. No growth was detected in the wells containing either antibiotics even at the final day, day four, of measurements.

**Table 6:** Effects of CCCP and EDTA on the MIC level of kanamycin and gentamycin.

	Kanamycin			Gentamycin		
		
Family/Genus:	MIC (mg/L)	MIC in presence of 50 μM CCCP	MIC in presence of 1mM EDTA	MIC (mg/L)	MIC in presence of 50 μM CCCP	MIC in presence of 1 mM EDTA
*Achromobacter*	>256	>256	>256	128	128	64
*Arcicella*	NG	NG	NG	NG	NG	NG
*Chitinophaga*	>256	CI	CI	>256	CI	CI
*Chryseobacterium*	>256	>256	>256	>256	>256	>256
*Comamonas*	32	32	8	>256	>256	64
*Ferruginibacter*	NG	NG	NG	NG	NG	NG
*Flavihumibacter*	128	CI	CI	16	8	CI
*Flavobacterium*	128	CI	CI	32	32	CI
*Flavobacterium*	>256	>256	<4	>256	>256	>256
*Flavobacterium*	NG	NG	NG	NG	NG	NG
*Flavobacterium*	NG	NG	NG	128	32	32
*Granulicella*	>256	>256	>256	>256	>256	>256
*Heliimonas*	64	CI	64	32	CI	8
*Herbaspirillum*	>256	>256	128	>256	>256	>256
*Micrococcus*	4	CI	CI	256	256	CI
*Mucilaginibacter*	>256	32	>256	64	64	128
*Mycobacterium*	32	32	4	64	32	<4
*Pannonibacter*	>256	128	64	<4	<4	<4
*Parasediminibacterium*	NG	NG	NG	NG	NG	NG
*Pedobacter*	>256	>256	128	>256	>256	32
*Pedobacter*	>256	>256	>256	>256	>256	>256
*Pedobacter*	64	64	32	>256	>256	64
*Pedobacter*	64	64	32	128	128	64
*Pedobacter*	64	32	CI	>256	>256	CI
*Phreatobacter*	128	CI	CI	64	CI	CI
*Polaromonas*	64	CI	CI	32	CI	CI
*Reyranella*	16	<4	<4	32	CI	4
*Xanthobacteraceae*	>256	>256	>256	>256	>256	>256
*Escherichia coli* (negative control)	<4	<4	<4	<4	<4	<4


*NG = no growth in MH medium control without additions. CI = CCCP and/or EDTA inhibited growth completely*.

### Evolutionary Relationship of Proposed Mechanism of Resistance

**FIGURE 4:**
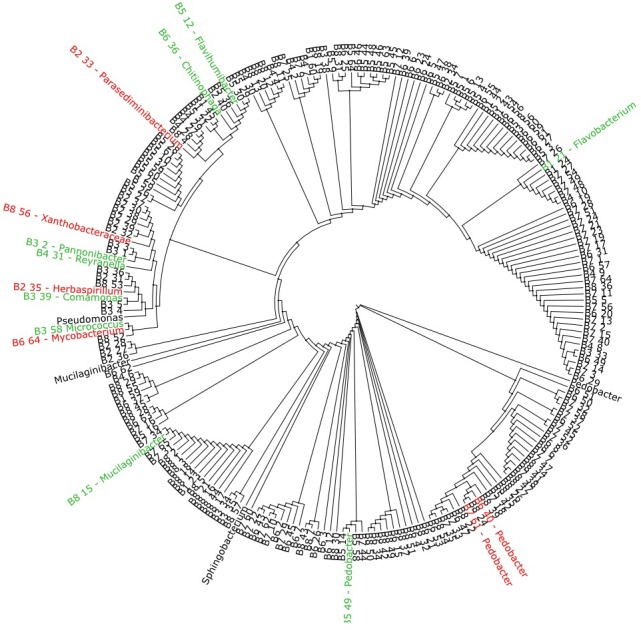
Phylogenetic tree based on 16S rRNA amplicon sequencing of 223 aminoglycoside resistant isolates. The isolates marked in red where found to harvest one or more of the selected AME genes screened for with multiplex-PCR (Table 4), while the isolates marked in green showed a reduction in MIC value when exposed to either CCCP or EDTA (Table 6). The marked isolates (both green and red), branch out on 12 different clades.

Based on the 16S rRNA amplicon sequencing data, a phylogenetic tree of the aminoglycoside resistant isolates was produced (Figure 4). The 15 isolates where a mechanism of resistance was proposed branch out on 12 different clades and represent 13 different genera or families. The isolates in which one or more of the selected AME genes were detected are marked in red, while isolates that had a reduction in MIC value when exposed to CCCP or EDTA are marked in green.

## Discussion

The major objective of this study was to identify aminoglycoside resistant bacteria present in sludge produced at DWTPs in Norway, and to describe, where possible, their resistance mechanisms. The findings provide information on the numbers and taxonomic diversity of the aminoglycoside resistant bacteria in each sample and, by extension, some information on the reservoirs of determinants of aminoglycoside resistance at the sample sites. Sludge samples are formed by the removal of organic material from the raw water entering the DWTP. This material represents both the water source and run off from the soil catchment area. Bacteria present in sludge produced at DWTPs may, therefore, represent the taxonomic diversity of large areas with limited anthropogenic activity.

Sludge from 23 DWTPs at different geographical locations in Norway was sampled and analyzed. Bacteria resistant to kanamycin were observed in 65% of all samples, while 68% of the DWTP samples gave growth of gentamycin resistant bacteria. These results indicate that resistance to clinically important AG is wide-spread in the environmental samples investigated. Bacterial cultivation showed that in 30% of the samples, kanamycin resistant bacteria made up more than 10% of the total colony count. The same trend was observed for gentamycin. The largest difference between the numbers of kanamycin and gentamycin resistant bacteria was 20% (DWTP 15). Therefore, resistance to one or the other antibiotic does not appear to be overrepresented in the majority of DWTP sludge samples. For the DWTPs where results were available for both kanamycin- and gentamycin-resistant isolates, no significant difference in CFU/mg sludge were observed (Table 3). This may indicate common, perhaps intrinsic, mechanisms of resistance. The results presented indicate that the aminoglycoside resistant bacteria in these environments make up a significant proportion of the total R2A plate count. Thus the effect of reintroducing these resistant isolates into the environment through the use of sludge as fertilizer is worthy of further evaluation.

The aminoglycoside resistant isolates investigated in this study, were mainly taxa commonly associated with either soil or fresh water (Woegerbauer et al., 2015; Narciso-da-Rocha and Manaia, 2016; Viana et al., 2018). Furthermore, the populations of bacteria found at the sample sites have a similar taxonomic structure, e.g., bacteria belonging to the phylum *Bacteroidetes* were dominant in all but one sample. Of these, the three most abundant genera detected were *Pedobacter, Flavobacterium* and *Mucilaginibacter.* Out of the 223 where the 16S rRNA genes were successfully sequenced, 25% belonged to the genus *Pedobacter*. This finding seems to be particularly important. A recent study describes *Pedobacter* spp. as environmental superbugs with multidrug antibiotic resistance. What appears to be mainly intrinsic resistance in this genus, covers not only aminoglycosides, but also ß-lactams and ciprofloxacin. Preliminary data indicate that several of the *Pedobacter* isolates from the present study show the same resistance trends observed by Viana et al. (2018) We are currently attempting to identify the resistance determinants in a number of sludge associated *Pedobacter* isolates by whole genome sequencing.

Multiplex-PCR screening showed low occurrences of the four target genes coding for AMEs, indicating that the majority of the observed resistance to kanamycin and gentamycin in this study is not due to the presence of these enzymes. One of the target genes, *aac(3*′*)-II*, was not detected at all in the present study. The low levels of AME genes detected is in accordance with a study performed in Northern Norway, where none of the 2000 kanamycin resistant isolates from soils and feces on pig farms were positive for *aph(3*′*)-III (nptIII)* or *aph(3*′*)-II (nptII*) (Nordgård et al., 2016). A report from maize fields in Austria also concluded that the prevalence of *aph(3*′*)-II* (*nptII*) and *nptIII* was low (Woegerbauer et al., 2015). *aph(3*′*)-II (nptII)* was only detected in *Pedobacter*. Woegerbauer et al. (2015) reported that *nptIII* was detected in *Pedobacter cryoconitis* and *Mucilaginibacter* sp. isolates. It is important to note that there are several other aminoglycoside modifying enzymes that were not included in this study, and further work could assay for other ARG of clinical relevance. The results suggests that AG-resistance genes are present in Norwegian drinking water sludge samples, but at low concentrations. Although limited to testing of four ARG, it seems unlikely that the measured resistances of 223 isolates are mainly a result of classic, single-gene based mechanisms which might be transferable to clinically relevant isolates. As such, the situation in Norway seems promising at the time of writing.

To investigate whether the action of efflux pumps and/or the outer membrane could account for the observed resistance to aminoglycosides in isolates which did not possess ARG, a modified Broth microdilution assay was performed. Both the membrane permealizer EDTA and the protonophore CCCP were found to affect MIC values of several of the tested isolates. The MIC assay was based on the CLSI standard M100, but modified to fit the growth characteristics of environmental isolates (e.g., lower temperature and longer incubation times). The inclusion of the CLSI control strain *E. coli* ATCC 25922, showed that the aminoglycosides used in this assay were stable throughout the course of the experiment.

Taken together, the results of the present study indicate that for some isolates the observed resistance to kanamycin and/or gentamycin could be a result of AMEs, protection by the outer membrane or by activation of efflux pumps. A limitation of the CCCP-inhibition tests is that the agent is a protonophore, which can have multiple effects on the cell, including increases in permeability, reduction in ATP production and changes in transmembrane potential/proton motive force (Morita et al., 1998; Osei Sekyere and Amoako, 2017). This makes direct interpretation of the inhibitory effects seen in the experiment difficult. Similarly, EDTA as cation chelator may also have multiple effects on the cell (Finnegan and Percival, 2015). Furthermore, the present study uses single concentrations of CCCP (50 μM) and EDTA (1 mM). It would be useful in future work to preform dose-response curves using a range of concentrations.

The phylogenetic tree of the 223 AG-resistant isolates shown in Figure 4, shows their relative taxonomic placements and highlights those where a resistance mechanism was detected in this study. The 15 isolates highlighted represent 13 genera or families showing that the proposed resistance mechanism do not map to one or a small number of taxa present in the sludge samples. Taken together, the results suggest AG-resistance is widespread in sludge. Although most isolates were Gram-negative bacteria, resistance is not in the domain of a limited number of taxa. For most isolates, however, a mechanism of resistance was not identified. Other important intrinsic and acquired mechanisms, e.g., methylases, exist which were not included in this study (Vaz-Moreira et al., 2014). Further work using techniques such as shotgun metagenomics sequencing, qPCR and whole genome sequencing (which is underway in our laboratory) could be used to gain more information on resistance to aminoglycosides.

## Conclusion

In conclusion, this study provides for the first time information on the presence and distribution of aminoglycoside resistance genes and bacteria in sludge from Norwegian DWTPs. If indeed AG-resistance genes are present at only very low concentrations in sludge samples, as this study indicates, any addition to the soil/water from which the sludge is derived, might be considered as a significant environmental pollution with potential effects on the involved ecosystems (Woegerbauer et al., 2015). Taken together, the results of the present study suggest a low selective pressure for the development of aminoglycoside resistance in the Norwegian environment. This might be a reflection of the moderate levels of aminoglycoside in use in Norway and present restrictions on GMO.

## Author Contributions

IU contributed conception and designed the study, performed the lab work and wrote the first draft of the manuscript. HT and CC contributed conception and designed the study, analyzed and interpretation of data and wrote sections of the manuscript. CC was the main coordinator of the study. HT contributed in the lab. MA and VL developed the protocol for and organized to collected the sludge samples. MA wrote sections of the manuscript. KN contributed with analysis and interpretation of data. All the authors contributed to manuscript revision, read and approved the submitted version.

## Conflict of Interest Statement

The authors declare that the research was conducted in the absence of any commercial or financial relationships that could be construed as a potential conflict of interest.
